# Processing Surface EMG Signals for Exoskeleton Motion Control

**DOI:** 10.3389/fnbot.2020.00040

**Published:** 2020-07-14

**Authors:** Gui Yin, Xiaodong Zhang, Dawei Chen, Hanzhe Li, Jiangcheng Chen, Chaoyang Chen, Stephen Lemos

**Affiliations:** ^1^Institute of Robotics and Intelligent Systems, School of Mechanical Engineering, Xi’an Jiaotong University, Xi’an, China; ^2^Shaanxi Key Laboratory of Intelligent Robots, Xi’an Jiaotong University, Xi’an, China; ^3^Robotic Rehabilitation Laboratory, Department of Biomedical Engineering, Wayne State University, Detroit, MI, United States; ^4^Shenzhen Academy of Robotics, Shenzhen, China; ^5^Department of Rehabilitation Medicine, First Affiliated Hospital, Fujian Medical University, Fuzhou, China; ^6^Department of Orthopaedic Surgery and Sport Medicine, Detroit Medical Center, Detroit, MI, United States

**Keywords:** exoskeleton, gait, electromyography, volitional control, treadmill, rehabilitation

## Abstract

The surface electromyography (sEMG) signal has been used for volitional control of robotic assistive devices. There are still challenges in improving system performance accuracy and signal processing to remove systematic noise. This study presents procedures and a pilot validation of the EMG-driven speed-control of exoskeleton and integrated treadmill with a goal to provide better interaction between a user and the system. The gait cycle duration (GCD) was extracted from sEMG signals using the autocorrelation algorithm and Bayesian fusion algorithm. GCDs of various walking speeds were then programmed to control the motion speed of exoskeleton robotic system. The performance and efficiency of this sEMG-controlled robotic assistive ambulation system was tested and validated among 6 healthy volunteers. The results demonstrated that the autocorrelation algorithm extracted the GCD from individual muscle contraction. The GCDs of individual muscles had variability between different walking steps under a designated walking speed. Bayesian fusion algorithms processed the GCDs of multiple muscles yielding a final GCD with the least variance. The fused GCD effectively controlled the motion speeds of exoskeleton and treadmill. The higher amplitude of EMG signals with shorter GCD was found during a faster walking speed. The algorithms using fused GCDs and gait stride length yielded trajectory joint motion tracks in a shape of sine curve waveform. The joint angles of the exoskeleton measured by a decoder mounted on the hip turned out to be in sine waveforms. The hip joint motion track of the exoskeleton matched the angles projected by trajectory curve generated by computer algorithms based on the fused GCDs with high agreement. The EMG-driven speed-control provided the human-machine inter-limb coordination mechanisms for an intuitive speed control of the exoskeleton-treadmill system at the user’s intents. Potentially the whole system can be used for gait rehabilitation of incomplete spinal cord hemispheric stroke patients as goal-directed and task-oriented training tool.

## Introduction

Robotic ambulatory exoskeletons for physical rehabilitation have been utilized for spinal cord injury (SCI) and stroke patients’ rehabilitation to enhance motor recovery in recent years. Interactive control of a rehabilitative robotic assistive device with the patient intention resulted in better clinical treatment outcomes (Jin et al., 2014; Zhang et al., 2018). There are several human-robot interactive control strategies for a rehabilitation robot including trajectory control and impedance control (Hussain et al., 2011). Trajectory control guides the exoskeleton to move on fixed trajectory tracks (Lum et al., 2002; Emken et al., 2007) and does not provide volitional control of the exoskeleton motions. Impedance control mechanisms (Wen et al., 2011) adjust the robotic stiffness, actuator force and position to get a designated interaction. This impedance control has been used in the Lokomat robot for rehabilitation training among patients (Jezernik et al., 2004; Maggioni et al., 2018), but the impedance parameters need to be manually adjusted for different patients making it difficult to select the appropriate impedance parameters (Riener et al., 2005). These researches demonstrated a trend that neurorehabilitation technologies have been directed toward creating robotic exoskeletons to restore motor function in impaired individuals. However, current robotic exoskeletons have had only modest clinical impact due to the inability to enable exoskeleton voluntary control in neurologically impaired individuals. This hinders the possibility of optimally inducing the activity-driven neuroplastic changes for a better function recovery (Durandau et al., 2019).

Bio-electrical signal control methods for robot-assisted rehabilitation have showed positive and promising outcomes with moderate evidence of improvement in walking and motor recovery using robotic devices compared to traditional practice (Soekadar et al., 2011, 2015; Hobbs and Artemiadis, 2020). A robotic assistive device with volitional control of walking speed may produce better rehabilitation outcomes (Manurung et al., 2010). The patients with disability desire to have the ability to control their walking speed with assistance from a rehabilitative robotic system. This can encourage a patient to actively engage in rehabilitation training (Koenig et al., 2009; Zhang et al., 2018).

The real-time volitional control algorithms using electromyogram (EMG) signals can be an optimal way to achieve harmonic interactions between the user and the robotic assistive ambulatory device. EMG signals have been used to control assistive rehabilitative devices (Kiguchi and Hayashi, 2012; Tong et al., 2014; Young et al., 2017; Choi et al., 2018; Gandolla et al., 2018; Durandau et al., 2019). However, machine motion control based on EMG signal can be affected by signal-noise-ratio (SNR) and artifacts (Gwin and Ferris, 2012). There are challenges of bioelectrical signal processing including removal of systematic noise, identification of EMG unrelated to walking, overcoming signal variability, classification algorithm robustness, and quantifiable performance feedback indicators (Tariq et al., 2018). These challenges are required to be solved for the methods to become viable parts of rehabilitation, especially in exoskeleton implementations (Ison and Artemiadis, 2014).

Assistive exoskeleton device control techniques include assist-as-needed (AAN) control using a model predictive control approach (Teramae et al., 2018) and EMG based impedance control for assistive exoskeletons (Kiguchi and Hayashi, 2012). Unfortunately, the motion speeds of these control methods are not adjustable.

The walking speed can be determined by a gait stride distance over a gait cycle duration (GCD) (Ardestani et al., 2016). A gait cycle is defined as the interval of time between two repetitive events of walking. One full gait cycle begins at the heel strike of one foot and continues until the heel strike of the same foot in preparation for the next step. The average duration of one gait cycle for men ranges from 0.98 to 1.07 second (Murray et al., 1964; Mark Burden et al., 2014). The speed should be precisely controlled by GCD when a gait stride distance is constant. We hypothesized that GCD can be extracted from EMG signals for machine motion speed control. Using EMG signal and derived gait cycle duration (GCD) to control an assistive ambulation system and a treadmill simultaneously has not been reported.

Many of the exoskeletons also use a treadmill as a combined system for training in order to keep certain variables consistent, such as average walking speed. The treadmill allows for the execution of walking cycles in a relatively small and controlled space (Hesse, 2008). Training with a treadmill has improved the long-term effects in post-stroke (Reisman et al., 2005). Methods for treadmill speed control have been reported. Minetti et al. (2003) measured the patient’s position on a treadmill and used a proportion-integral-derivative (PID) control algorithm to calculate the treadmill velocity. Treadmill velocity control can be implemented by using the ground reaction force between the subject’s foot and the ground (Von Zitzewitz et al., 2007; De Luca et al., 2009; Feasel et al., 2011). An algorithm was optimized to limit the maximum acceleration and balance the treadmill speed (Souman et al., 2010). Liu et al. (2015) used pressure sensors mounted on the treadmill to obtain the force signal and determine the acceleration and deceleration of the subject’s movements. In a combined system including exoskeleton ambulation assistive system and a treadmill, exoskeleton walking velocity should be the same speed of treadmill moving speed, as long as GCD can be used for exoskeleton speed control, it also can be sued for treadmill speed control.

The motivation of our research is to use voluntary-controlled task-orientated robotic device for clinical rehabilitation of stork and incomplete SCI, hopefully to yield better clinical function recovery. Because the SNR (signal-to-noise ratio) is one of major concerns for signal processing and system performance, one of our motivations is to discover a better method of EMG signal processing with less artifact and noise. We have developed a prototype of a rehabilitative exoskeleton integrated with a customized treadmill. sEMG signal processing methods and algorithms to simultaneously control both exoskeleton and treadmill motions. The aims of this research were to develop novel EMG signal processing algorithms for volitional control an exoskeleton and integrated treadmill and to remove artifacts and systematic noise. We investigated the EMG-derived GCD extraction methodologies and tested efficiency of GCD in exoskeleton and treadmill motion velocity control at user’s intent when gait stride was set as a constant parameter. Real-time GCDs were obtained using autocorrelation algorithm with fusion algorithm. The performance of whole system was validated among health subjects. Potentially, our methods can be used to process weak remnant EMG signals in incomplete SCI and stroke patients for their rehabilitation trainings.

## Materials and Methods

### System Overview

**FIGURE 1:**
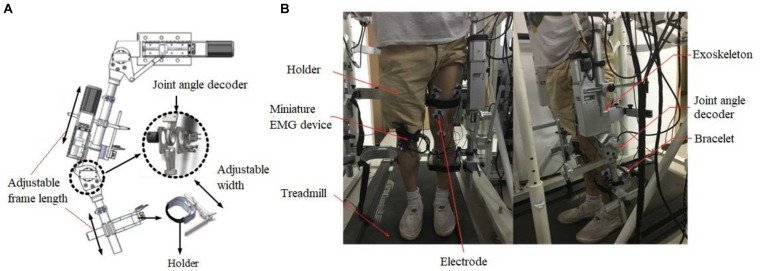
The illustration of assistive exoskeleton ambulation system. **(A)** The main components of exoskeleton showing adjustable frame and joint angle decoder. **(B)** The setup of the whole system showing exoskeleton and treadmill and the location of EMG electrode placement.

Our EMG controlled robotic assistive ambulation rehabilitation system includes a lower limb exoskeleton, EMG sensors and signal processing system, a treadmill, and a motion control software and firmware. The lengths of exoskeleton thigh and calf sections were adjustable to fit patients with different heights. Servo motors were used as the actuators to drive the knee and hip joint in the exoskeleton system. The Intrepid MTC-2.2A treadmill was used and integrated with the exoskeleton (Figure 1). Position encoders were installed at knee and hip joints to track the joint angle.

### Surface EMG Signal Recording and Processing

An 8-channel wireless EMG acquisition device (Nuocheng Electronic Company, Shanghai, China) was used for downstream EMG signal recording with dry copper electrodes. Surface EMG (sEMG) signals were recorded from 5 lower limb muscles, including: the vastus medialis (VM), vastus lateralis (VL), sartorius (SR), tensor fascia lata (TL), and soleus (SL). The sample rate was set at 1000 Hz. The sEMG signals were filtered by a 50 Hz (electrical power noise frequency) notch filter and a 20 Hz zero-lag fourth-order recursive Butterworth filter and then rectified. The magnitude of sEMG signals was measured by using the root mean square (RMS) method (Equation 1). Time window length of sliding/moving RMS method was set at 0.1 s (Mark Burden et al., 2014) for calculation of EMG RMS.

### Extraction of GCD From EMG Signals

**FIGURE 2:**
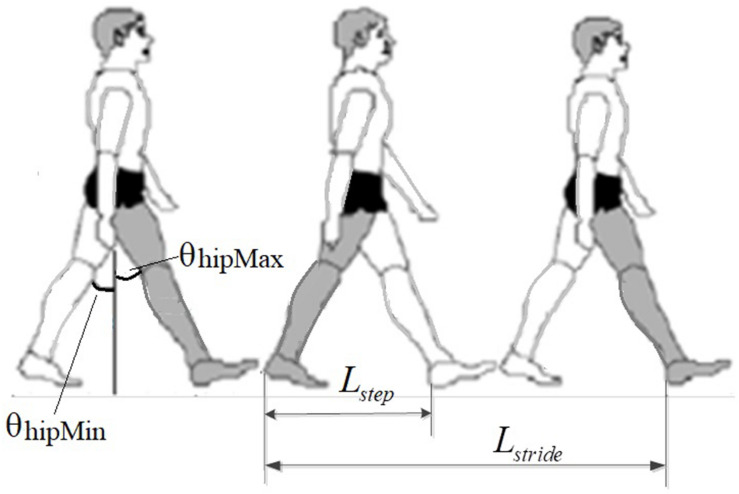
Measurement of stride length in walking, showing the right foot stride length. The hip joint angle of a full stride was constant. The change of GCD caused speed changes.

The EMG-derived GCD in this study was defined as the duration between the heel-lifting ground and heel landing on the ground (Murray et al., 1964) (Figure 2). The GCD measured from the right foot stride was used for speed control of both left and right legs alternately. Because subjects had different height leading to different stride length, the hip joint angle (θ) of a full stride was kept constant during exoskeleton walking, only EMG-derived GCD changed corresponding to muscle contraction duration (Figure 2).

The GCD was measured using two methods: the first method was the measurement of onset-to-onset time interval between two adjacent autocorrelation curves (Figure 5C), the second method was the peak-to-peak interval between two adjacent autocorrelation curves (Figure 5C). The first GCD measurement was synergized with the duration between the heel-lifting ground and heel landing on the ground. The second measured GCD was used in computer algorithms for machine velocity control.

### Autocorrelation and Bayesian Redundant Fusion Algorithms

Each muscle contraction yielded a specific autocorrelation curve. The RMS of sEMG voltage was obtained and processed using an autocorrelation and Bayesian fusion algorithms to extract the GCD. Formula for RMS calculation is shown in Equation 1.

(1) $$x_{R\,M\,S}=\sqrt{\frac{1}{N}\,\sum_{i=1}^{N}x_{i}^{2}}$$

Where *x*_*RMS*_ represents the RMS value of the sEMG signal, *N* represents the window length, and *x*_*i*_ is the instantaneous amplitude value of a sEMG voltage reading. The 50 and 80 ms window lengths showed undulating envelopes. A window length greater than 100 ms made the system sluggish. The optimum window length of 100 ms was chosen.

The autocorrelation function is shown in Equation 2:

(2) $$R_{x}\,\left(t_{1},t_{2}\right)=E\,\left[x\,\left(t_{1}\right)\,x\,\left(t_{2}\right)\right]=\int_{-\infty }^{\infty }\int_{-\infty }^{\infty }x_{1}\,x_{2}\,f_{x}\,\left(x_{1},x_{2};t_{1},t_{2}\right)\,dx_{1}\,dx_{2}$$

Where *R*_*x*_(*t*_1_,*t*_2_) represents the autocorrelation value of the RMS for sEMG signal *x*. *x*_1_ and *x*_2_ represent the RMS of sEMG signals at time *t*_1_ and *t*_2_. *f*_*x*_(*x*_1_,*x*_2_;*t*_1_,*t*_2_) represents the probability density function of the sEMG signals with respect to time.

The autocorrelation algorithm continuously calculated the correlation value of RMS associated with the gait cycle at each sampling point to generate autocorrelation curves. There is a peak point of the autocorrelation curve during each gait cycle, regardless of the initial gait phase. Duration between adjacent peak points of autocorrelation curve is defined as GCD (Lihong et al., 2010) (Equation 3).

(3) $$T=t_{s\,E\,M\,G\,\left(i\right)}-t_{s\,E\,M\,G\,\left(i-1\right)}$$

Where T represents the extracted GCD, *T* = *t*_*sEMG*(*i*)_ and *t*_*sEMG(i–1)*_ represent the time of the i-th peak point and the time of the [(i-th)-1] peak point, respectively.

The final GCD was obtained by averaging individual GCDs from five muscle sensors using the Bayesian redundant fusion algorithm. The sEMG sensor was modeled as a Gaussian distribution function with a mean about the true value and a variance about the uncertainty due to noise. The GCD extracted by the single-channel probabilistic sensor is shown in Equation 4:

(4) $$p\,\left(T_{j}|y\right)=\frac{1}{\sigma_{j}\,\sqrt{2\,\pi }}\,e^{\frac{-{\left(y-T_{j}\right)}^{2}}{2\,\sigma_{j}^{2}}},j=1,2,\ldots ,5$$

Where *p*(*T*_*j*_|*y*) is the probability distribution of each single-channel sEMG sensor, σ_*j*_ is the standard deviation of each single sEMG sensor, and they are: σ_1_ = 1.58, σ_2_ = 0.96, σ_3_ = 2.91, σ_4_ = 0.91, σ_5_ = 1.18, respectively. *y* represents the true value of the gait cycle, *T*_*j*_ is the GCD extracted by each single sEMG sensor, and *j* denotes each different sEMG channel.

Bayesian redundant fusion was used to process the selected gait cycle datasets. The fused GCD was the mean of the fusion distribution function. The mean and variance of the fusion distribution function were expressed as, and shown in, Equations 5 and 6:

(5) $$\sigma_{f\,u\,s}^{2}=\frac{1}{\sigma_{1}^{-2}+\sigma_{2}^{-2}+\sigma_{3}^{-2}+\sigma_{4}^{-2}+\sigma_{5}^{-2}}$$

(6) $$\begin{array}{c} T_{f}={\text{μ}}_{fus}={\text{σ}}_{fus}^{2}(\frac{T_{1}}{{\text{σ}}_{1}^{2}}+\frac{T_{2}}{{\text{σ}}_{2}^{2}}+\frac{T_{3}}{{\text{σ}}_{3}^{2}}+\frac{T_{4}}{{\text{σ}}_{4}^{2}}+\frac{T_{5}}{{\text{σ}}_{5}^{2}})\\ =\frac{{\text{σ}}_{1}^{-2}T_{1}+{\text{σ}}_{2}^{-2}T_{2}+{\text{σ}}_{3}^{-2}T_{3}+{\text{σ}}_{4}^{-2}T_{4}+{\text{σ}}_{5}^{-2}T_{5}}{{\text{σ}}_{1}^{-2}+{\text{σ}}_{2}^{-2}+{\text{σ}}_{3}^{-2}+{\text{σ}}_{4}^{-2}+{\text{σ}}_{5}^{-2}} \end{array}$$

Where $\sigma_{f\,u\,s}^{2}$ and μ_*fus*_ represent the variance and the mean of the fusion distribution, respectively. *T*_*f*_ is the fused GCD.

In summary, After GCD curve was generated by autocorrelation algorithms, computer algorithms then determined the peak-to-peak time reading to generate a measured GCD. Bayesian fusion algorithm generated a fused GCD from multiple EMG channels. The final fused GCD was used for servo motor speed rotation speed control.

### Determination of Treadmill Motion Speed for Volitional Control

**FIGURE 3:**
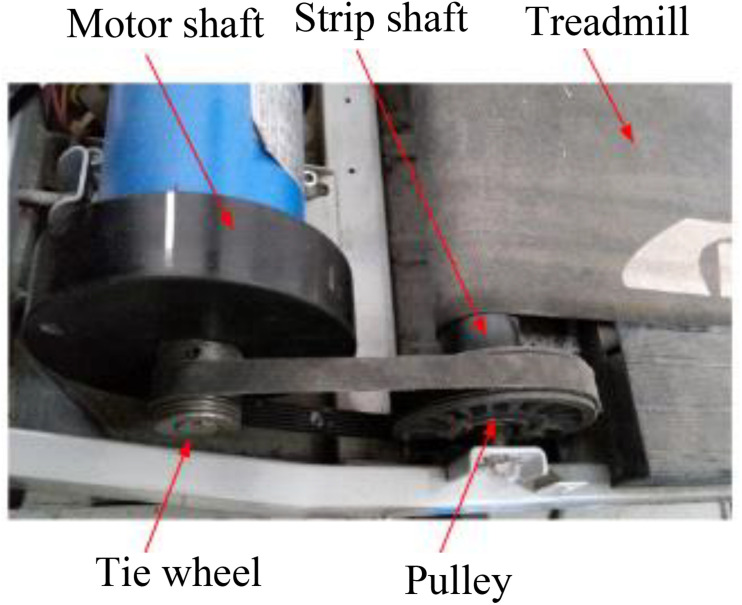
Shows a modified treadmill driver profile; servo motor was installed to drive the treadmill.

The exoskeleton and treadmill motion speed were determined by stride length over GCD (Figure 3). In this study, the stride length was a constant over walking, while GCD changed according to EMG signals. The shorter GCD produced a faster motion of machine. The machine movement velocity of the first two steps was set at default 0.4 m/s. The subject contracted muscle to follow the machine. The second step, the subject controlled the muscle contraction duration at subject’s intent during walking. Computer calculated the GCD between these two muscle contractions for the next step’s movement velocity control.

The stride length formula is shown in Equation 7:

(7) $$L_{G}=2\,\left(l_{h}+l_{k}\right)\,\left[\sin\left(\theta_{\text{hamax}}\right)-\sin\left(\theta_{\text{hamin}}\right)\right]$$

Where *L*_*G*_ represents the subject’s stride length, *l*_*h*_ represents the subject’s thigh length, *l*_*k*_ represents the subject’s calf length, and θ_*hamax*_ and θ_*hamin*_ represent the maximum and minimum hip angle, respectively.

The exoskeleton’s walking speed was calculated by dividing the stride length divided from the fused GCD. The treadmill speed was set at the same speed of exoskeleton walking speed by setting treadmill belt moving distance same as the stride length over the same GCD.

(8) $$V_{g\,a\,i\,t}=\frac{L_{G}}{T_{f}}$$

Where *V*_*gait*_ represents the subject’s walking speed, and *T*_*f*_ represents the fused GCD.

The treadmill speed was set at the same as the exoskeleton walking speed in the opposite direction, this can be expressed as shown in equation 9:

(9) $$V_{t\,r\,e\,a\,d}=-V_{e\,x\,o}=-V_{g\,a\,i\,t}$$

Where *V*_*tread*_ represents the treadmill speed, and *V*_*exo*_ represents the speed of the exoskeleton.

The treadmill motor drove the treadmill through the motor shaft and the pulley. The treadmill velocity was controlled by the rotation speed of a servo motor (Figure 3). The outer diameters of servo motor shaft and treadmill pulley were constant with a fixed ratio. Accordingly, the treadmill motor speed was controlled based on the following equation:

(10) $$V_{m}=\frac{V_{t\,r\,e\,a\,d}}{K\,\pi \,d_{t\,r\,e\,a\,d}}$$

Where *V*_*m*_ is the driving motor speed, *V*_*tread*_ is the treadmill velocity, *K* is the speed ratio of the treadmill motor to the pulley, π = 3.14, and *d*_*tread*_ is the outer diameter of the strip shaft (Figure 3).

### Exoskeleton Walking Velocity Control Mechanisms

**FIGURE 4:**
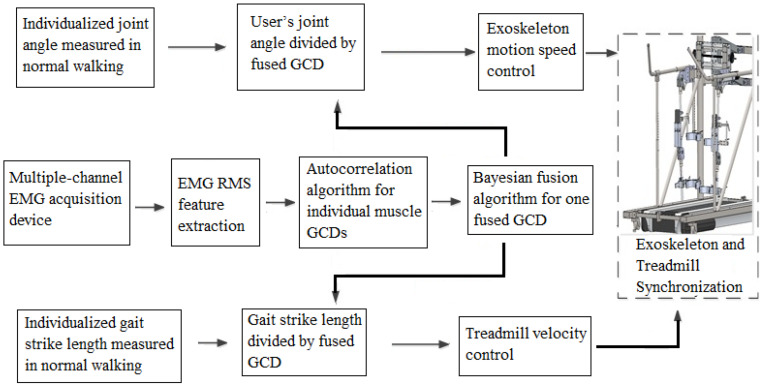
The schematic flowchart of EMG-controlled exoskeleton and treadmill system.

The range of motion (ROM) of the joints were set at a fixed degree from normal gait angle reference trajectory, EMG-derived GCD determined the angular speed of joints and the consequent walking speed. Shorter GCD yielded a faster walking speed. Close loop servo motors were used for exoskeleton and treadmill actuation. The speed of servo motors of exoskeleton and treadmill was controlled by pulse width modulation (PWM) output by microcontrollers (PMAC Clipper, Delta Tau Company, Beijing, China) based on obtained GCD. Frequency of out pulses was determined by GCD to control the rotation speed of servo motors. Computer C language was used to compile files, Matlab software was used to execute the controlling commands. PMAC Clipper is a multi-axis motion controller. Subject’s muscle contraction duration determined GCD and consequent machine motion speed. The exoskeleton joint angular speed was generated in the host controller by the sEMG based volitional control and then it was sent to the PMAC Clipper, which is a multi-axis motion controller (Delta Tau Company, Beijing, China) (Figure 4).

### Experimental Protocol for System Performance Validation

Six healthy subjects (3 males and 3 females, ages 23–32 years old, 1.70 ± 0.14 m in height (Mean ± SD) and weight 61.6 ± 5.4 kg) were recruited to participate the system performance validation testing. All procedures have been approved by the Medicine Biomedical Ethics Committee in Xi’an Jiaotong University.

Warming-up exercise was performed by subjects to get them familiar with the whole system. Subjects were instructed to walk at a normal speed of approximately 1 m/s, with the starting position at the normal standing state. The sEMG signals were collected and the GCD was extracted from the sEMG signals to determine the accuracy of the GCD extraction algorithm. The speed was controlled by the subjects to be approximately 0.4, 0.6, and 1.0 m/s, respectively. The purpose was to obtain the peak values of autocorrelation curves under different speeds for writing codes in speed control algorithms.

The GCD extracted from sEMG signals was used to control the machine movement speed. Angular speed obtained from the decoder mounted at the hip joint was recorded. The GCD recorded at real-time was compared with the GCD obtained from EMG to measure the agreement between two readings to determine the accuracy of system performance.

Then the subject walked on the treadmill by wearing the exoskeleton under different speeds from slow (0.4 m/s) to fast (1 m/s) respectively for 10 min. During each trial, the zero degree of the knee and hip joints were set as the starting phase of the gait cycle at a preset speed of 0.4 m/s for the first step. User was not able to change the first step’s motion speed. However, user could change the muscle contraction duration to control the next step motion speed of exoskeleton and treadmill.

### Data Analysis

SPSS software (Version 25, IBM, Chicago, United States) was used for statistical analysis to determine the difference between measured outcomes by different methods. Reliability analysis was performed to determine the difference between the measured joint angle and algorithm predicted joint angle used for inter-rater agreement analysis. *P*-value smaller than 0.05 was considered to be a statistically significant level.

## Results

### GCDs for Normal Walking Speed

**FIGURE 5:**
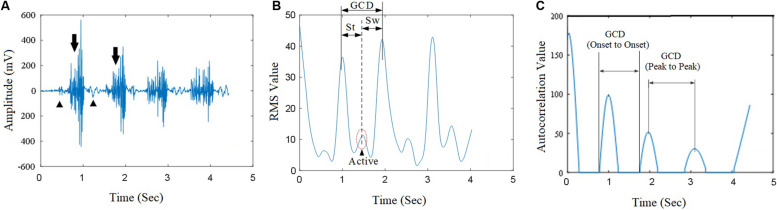
The raw sEMG signal from a subject’s vastus medialis (VM) and its autocorrelation value curve. **(A)** The raw sEMG signals corresponding with gait cycle during walking; **(B)** The corresponding EMG RMS curve; **(C)** The autocorrelation value extracted from the VM muscle. GCD was obtained based on the duration between two peaks of adjacent autocorrelation curves.

Muscle contractions during walking produced corresponding EMG signals (Figure 5A, arrows) and some noise signals (Figure 5A, arrow heads). True muscle contraction produced high amplitude of RMS curves while the amplitude of the noise’s EMG RMS curves was much smaller (Figures 5A,B). Raw EMG signal processing after RMS calculation yielded smooth curves corresponding to muscle contractions (Figure 5B). The muscle contractions yielded the characteristics of periodicity of the RMS of sEMG signals. The EMG signal processing method using autocorrelation algorithm yielded autocorrelation curves that clearly showed onset points and peak points of muscle activations. The autocorrelation curves were speared of the noise from EMG artifact and systemic noise. Signals from artifacts and unrelated muscle contractions did not fit into autocorrelation curve thus were removed from the autocorrelation curves.

**FIGURE 6:**
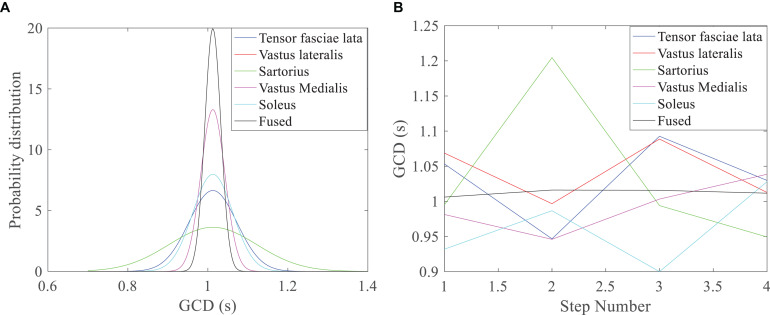
GCD profile of walking under the speed of 1 m/s. **(A)** Bayesian distribution GCD curves of 5 muscles in one step of walking; the fused GCD has the least SD (gray line), while GCD from the Sartorius has the largest SD (green line); **(B)** GCDs from different muscles, the Sartorius muscle has the higher variance over different steps (green line), while the fused GCD has the least variance (gray line).

The GCDs of individual muscles were different during a step of walking under a fixed walking speed (Table 1 and Figure 6A); the sartorius muscle had the largest variance or standard deviation of GCD measurement (green line, Figure 6A). The GCDs of individual muscles were different between different walking steps under a designated walking speed; the sartorius muscle had the largest variance of GCDs over the steps (green line, Figure 6B). Bayesian redundant fusion algorithms processed GCDs of all 5 muscles yielding a GCD with the least variance of GCD in one step of walking (Figure 6A, gray line) and over the steps of walking (Figure 6B, gray line).

**TABLE 1:** Individual GCDs and fused GCD (Mean ± SD).

Channel	Subject 1	Subject 2	Subject 3	Subject 4	Subject 5	Subject 6
sEMG(VM)	1.103 ± 0.088	0.992 ± 0.039	1.053 ± 0.039	1.055 ± 0.100	1.131 ± 0.101	1.016 ± 0.032
sEMG(TL)	0.987 ± 0.031	0.977 ± 0.052	0.964 ± 0.062	0.991 ± 0.044	1.011 ± 0.039	0.971 ± 0.064
sEMG(VL)	0.900 ± 0.092	0.839 ± 0.0117	0.916 ± 0.100	0.882 ± 0.104	0.936 ± 0.081	0.938 ± 0.090
sEMG(SR)	1.028 ± 0.038	1.200 ± 0.131	1.163 ± 0.104	1.036 ± 0.050	0.998 ± 0.032	1.131 ± 0.118
sEMG(SL)	1.005 ± 0.027	1.002 ± 0.028	1.025 ± 0.030	1.011 ± 0.038	1.018 ± 0.030	1.021 ± 0.038
Fused	1.005 ± 0.010	1.002 ± 0.012	1.024 ± 0.017	0.998 ± 0.016	1.019 ± 0.018	1.015 ± 0.021

*Table 1 shows individual GCDs and fused GCD with walking speed of 1 m/s. The maximum variance of fused GCD obtained by using the multi-channel sEMG fusion algorithm for this walking speed ranged from 0.010 to 0.021 in this study.*

### Profile of Joint Angle Kinematics

**FIGURE 7:**
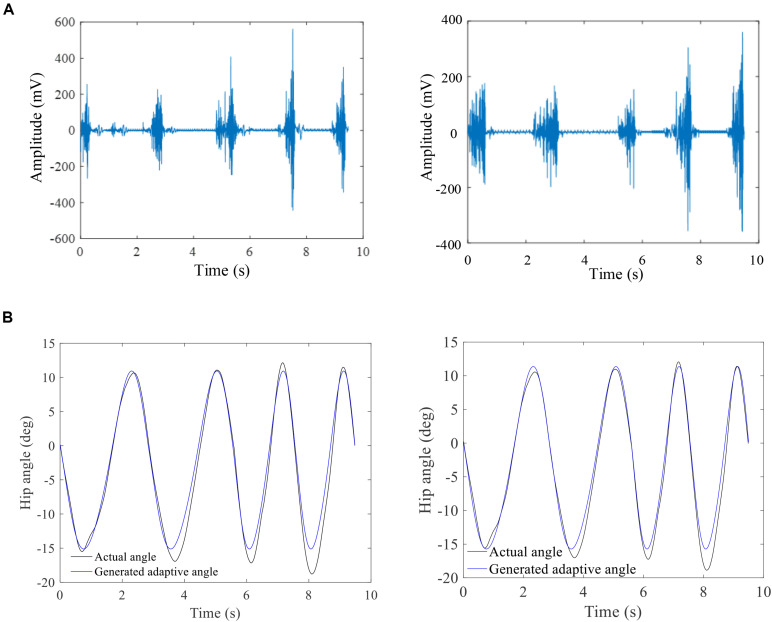
The raw sEMG and corresponding a hip joint angle profile. **(A)** Raw sEMG signals showing increase of EMG amplitude with shorter duration between two muscle contractions with increased walking speed; **(B)** the GCD-based computer-generated hip joint angles (blue line) matches the measured exoskeleton hip joint angles during walking (black line). The left column is the results of subject 1 and right column is the results of subject 6.

The results showed that the higher amplitude of EMG signals with shorter GCD was found during a faster walking speed (Figure 7A). The algorithms based on fused GCDs and gait strike length yielded trajectory joint motion track in a shape of sine curve (Figure 7B, black line). The hip joint angles of the exoskeleton measured by a decoder mounted the hip joint turned out to be in a track of sine waveforms. The exoskeleton hip joint motion waveform matched the angles projected by hip motion encoding curve based on GCD (Figure 7B) with high agreement (Reliability analysis, Cronbach value = 0.85, *p* = 0.001).

## Discussion

### Summary

This study demonstrated a novel approach to extract GCD from sEMG signals for exoskeleton motion speed control. The autocorrelation algorithm extracted GCDs from muscles that removed noise from EMG signals. Different muscles had different GCDs in a step of walking as well as over the different steps of walking. Bayesian fusion algorithm processed GCDs of multiple muscles to yield a fused GCD. The fused GCD was encoded to control the motion speeds of exoskeleton and treadmill yielding a volitional EMG-controlled exoskeleton integrated with a treadmill system.

### Significance of Processing EMG for Exoskeleton Motion Control

EMG signals have been used for robotic assistive motion control using the trajectory control mechanism (Kiguchi and Hayashi, 2012; Tong et al., 2014; Teramae et al., 2018). Unfortunately, the motion speeds of these control methods are not adjustable. Adjustable walking speed control can be used for task-oriented rehabilitation train to encourage patients into the rehabilitation training. EMG signal amplitude has been used to trigger trajectory motions along the designated tracks. Due to the fluctuated EMG signal baseline and artifact, unwanted motion of the robotic system could be accidently triggered. Hence the trigger threshold is set high, but the higher triggering threshold leads to system response insensitive. Autocorrelation approach has been studied for EMG signal processing (Enders and Nigg, 2015). This study demonstrated EMG-derived autocorrelation algorithm generated a corresponding GCD curve without being affected by noise or artifacts.

The GCDs of individual muscles were different due to bio-variability from different individuals. Bayesian fusion algorithms has been using in handling the problem of uncertainty and inconsistency of the data in both centralized and decentralized data fusion architectures (Waleed and Abdulhafiz, 2013). This study demonstrated that Bayesian fusion algorithms by combining data from several sources using multiple sensor data and fusion algorithms reduced signal uncertainty. The fused GCD removed the variance of GCDs from multiple muscles EMG recording, yielding a stable GCD that better reflected user’s walking speed intents. The maximum variance of fused GCD obtained using the multi-channel sEMG fusion algorithm was 0.021 s in this study, which was smaller than that reported in the literature (0.046 0.055 s) (Lihong et al., 2010). This indicates that the Bayesian redundant fusion algorithm is an effective signal processing method to obtain optimal GCD with less deviation.

To date, the joint motion speed is predefined and cannot be used to achieve variable velocity control based on subject’s intended motion (Teramae et al., 2018). It is difficult to get the harmonic interactions between subject and assistive ambulation system with volitional control. In our study, the joint motion speed was encoded by fused GCDs extracted from user’s muscle contraction generated EMG signals. The results demonstrated that exoskeleton walking speed could be adjusted by measuring the sEMG signals to adjust exoskeleton motion speed. Faster walking speed led to high amplitude of EMG signals and shorter GCDs hence increasing the exoskeleton motion speed by computer programming. This suggested that using fused GCD as a single variable to control exoskeleton walking speed is an effective method while keeping the angle range of joint motion unchanged.

EMG based approach is an alternative approach to the wearable sensor approach. In incomplete spinal cord injury (iSCI) patients, for example, patients with ASIA grade B and C severity, these patients have very weak muscle contraction which cannot lift limb against gravity. Wearable sensor approach (Hassan et al., 2014) may be difficult to detect force changes and joint angle changes among patients with ASIA grade B and C severity, but EMG signal can be detected. Under this circumstance, EMG based approach better fits the iSCI rehabilitation training requirement.

### Significance of Processing EMG for Treadmill Motion Control

In this study, EMG based modular control of treadmill mechanism was utilized for treadmill speed control synchronized with exoskeleton walking speed. EMG based modular control approach has been used for treadmill speed for running (Oliveira et al., 2016). Modulation of locomotor-like EMG activity has been found in patients with incomplete spinal cord injury and EMG based modular control of treadmill has been used SCI patient rehabilitation (Dobkin et al., 1995). Our study also demonstrated the feasibility of using EMG for modular control of both exoskeleton and treadmill with inter-machine coordination.

Exoskeleton integrated with a treadmill for rehabilitation training has been used clinically with a consistent walking speed. However, treadmill training can be used without use of any direct attachment of robotic device. The treadmill allows for the execution of many walking cycles in a relatively small and controlled space (Hesse, 2008). Rehabilitation training with a treadmill have showed to have improved long-term effects in post-stroke gait (Reisman et al., 2010).

### Clinical Relevance

Robotic-assisted exoskeleton and treadmill training systems have been used in clinical rehabilitation for patients with neurological disorders. Exoskeleton ambulation systems can in the lower limb movements during walking training for rehabilitation for improvement and better outcomes including rehabilitation of spinal cord injuries (SCI) (Ungerleider et al., 2002) and stroke (Moucheboeuf et al., 2020). Volitional-controlled assistive rehabilitation devices has been reported to yield better functional recovery among stroke patients (Bundy et al., 2017). The improvement of motor function in stroke patients is associated with accuracy of motion-intention-associated signal processing performance (Bundy et al., 2017). Residual electromyography activity in stroke patients with complete paralysis has been shown to be decodable, even in cases when the movement is not possible (Loopez-Larraz et al., 2018) and incomplete spinal cord injury patients (Escalona et al., 2020; Murray et al., 2020). Because a large proportion of severe stroke patients have residual EMG signals, this yields a direct and practical way to trigger a novel rehabilitation using robotic rehabilitation engineering techniques (Balasubramanian et al., 2018). Our study provides a novel approach of EMG signal processing for volitional control of exoskeleton system for rehabilitation. Because the walking speed can be controlled at user’s intent, it can be used as a tool for task-orientated rehabilitation training, hopefully yielding a better clinical neural function recovery.

### Limitations

Limitations of this study are that the systematic validation has not been performed clinically among SCI or stroke patients. EMG derived GCD can change exoskeleton walking speed. It appeared to be that there were not irregularities when autocorrelation and fusion algorithms in healthy subject. It is not clear about the effectiveness of this signal processing method in the patients of sever gait impairments, although it has been reported in conference that autocorrelation may be feasible for lower limb EMG signals for the initial evaluation of hemiparetic gaits (Wang, 2016). Our clinical investigation will be conducted soon. GCD based velocity control algorithm could only be applied to the next steps of walking speed control. The kinematics of joint angle was programmed in sine waveform which does not match the real lower limb joint angular kinematic profile.

## Conclusion

This paper presented a novel approach to extract GCD from sEMG signals for exoskeleton motion control. The autocorrelation algorithm and the Bayesian redundant fusion algorithm extracted GCDs which were encoded for the exoskeleton and treadmill motion speed control according to user’s intent. Systematic noise and unrelated sEMG signals were automatically removed from the commanding signals for machine operations. GCD-based sEMG-controlled algorithms provided an adaptive interaction between the exoskeleton assistive ambulation system and the user.

## Data Availability Statement

The datasets generated for this study are available on request to the corresponding author.

## Ethics Statement

The studies involving human participants were reviewed and approved by the Medicine Biomedical Ethics Committee at Xi’an Jiaotong University. The patients/participants provided their written informed consent to participate in this study.

## Author Contributions

GY, XZ, and HL designed the framework. GY and HL carried out the experiments. GY, XZ, and JC performed the theoretical analysis. GY, DC, and CC analyzed the results and prepared the figures and tables. GY, CC, and SL wrote part of the manuscript and provided the critical review of whole project. All the authors contributed to the article and approved the submitted version.

## Conflict of Interest

The authors declare that the research was conducted in the absence of any commercial or financial relationships that could be construed as a potential conflict of interest.
